# Wild edible plants used by communities in and around selected forest reserves of Teso-Karamoja region, Uganda

**DOI:** 10.1186/s13002-018-0278-8

**Published:** 2019-01-09

**Authors:** Samuel Ojelel, Patrick Mucunguzi, Esther Katuura, Esezah K. Kakudidi, Mary Namaganda, James Kalema

**Affiliations:** Department of Plant Sciences, Microbiology & Biotechnology, School of Biosciences, College of Natural Sciences, Makerere University, P.O. Box 7062, Kampala, Uganda

**Keywords:** Wild edible plants, Food scarcity, Forest reserves, Teso-Karamoja, Uganda

## Abstract

**Background:**

The consumption of wild plants is an ancient tradition which serves multiple purposes. Cognizant that Teso-Karamoja region is frequently affected by food scarcity and is not adequately surveyed for its flora, this study sought to establish an inventory and use of wild edible plants by the communities living in and around the forest reserves.

**Methods:**

Data was collected using semi-structured questionnaires administered to 240 respondents living in and around eight forest reserves between November 2017 and May 2018. One focus group discussion (8–12 members) per forest reserve and field excursions to collect the plant voucher specimens were also conducted. The data was analyzed using descriptive statistics, relative frequency of citation (RFC), and the factor of informants’ consensus (FIC).

**Results:**

A total of 100 plant species in 47 families were reported as edible. *Carissa spinarum*, *Strychnos innocua*, *Balanites aegyptiaca*, *Tamarindus indica*, and *Ximenia americana* presented the highest RFC, while the families Rubiaceae, Fabaceae, Anacardiaceae, Amaranthaceae, and Moraceae had more than five species each. Grasses (Poaceae) comprised only 1% of the edible species and trees 35%, while shrubs were the most important source of wild food (RFC = 0.47). The fruits contributed 63% while leaves (29%), seeds (9%), tubers (5%), and gum (1%). The fruits were considered as the most important use category (RFC = 0.78). Respondent homogeneity was none for gum but very high for seeds (FIC - 0.93). Only 36% of species are cooked, while 64% are eaten in raw. Harvesting is done rudimentarily by digging (5%), collecting from the ground (fruits that fall down) (13%), and plucking from mother plants (82%). Only 9% of the species were collected throughout the year, 27% in the dry season, and 64% in the rainy season. The consumption of these plants is attributed to food scarcity, spicing staple food, nutri-medicinal value, cultural practice, and delicacy.

**Conclusion:**

A high diversity of wild edible plant species exists in the forest reserves of Teso-Karamoja region. The shrubs and fruits are the most locally important life forms and use category, respectively. These edible plant species are important throughout the year because their consumption serves multiple purposes.

## Introduction

The consumption of wild edible plants is an ancient phenomenon which predates agriculture [1]. These plants offer various benefits and opportunities to communities; for example, they enable communities to cope with food scarcity [2–4]. This is also known as ecosystem-based adaptation (EbA) [5]. They hold great cultural significance to dependent communities [6]. In addition, wild edible plants increase the nutritional quality of rural diets for instance, micronutrients (vitamins and minerals) which are sometimes superior to those of domesticated varieties [7]. Some of them also contain genes that can be sought to improve the productivity of cultivars [8]. They also contribute to household incomes [9], thereby contributing to the attainment of sustainable development goal 1 on eradicating poverty.

The selection of plants for ethnobotanical use is anchored in a theory. The theories include among others the optimal foraging theory [10] and theory of non-random plant selection [11]. The former predicts that foraging organisms will balance the effort it took to search for and eat that food. In so doing, individuals will place high value on plants that yield more benefit per unit of foraging/processing time; as abundance of plants with higher value increases, plants with lower value will no longer be used and individuals should have a quantitative threshold to decide when a specific plant should be included or excluded [12]. The latter theory asserts that plant selection is not random because species in the same family share some characteristics inherited from common ancestors (evolutionary relatedness) which in turn influence their physiology and ultimately their ethnobotanical use.

The global population facing food and nutritional insecurity increased from 777 million in 2015 to 815 million in 2016 [13]. In the same report, the percentage of population in Sub-Saharan and East Africa that was chronically undernourished was estimated to be 22.7 and 33.9%, respectively. In Uganda, an estimated 10.9 million people of the 40 million people experienced acute food insecurity in 2017 with Teso and Karamoja among the most affected areas [4]. These statistics underline the fact that food scarcity is one of the most pressing problems facing humanity globally [14]. The scarcity of food is largely caused by intermittent rainfall patterns which cause crop failure [4], warfare, poverty, and landlessness [15]. Unfortunately, these factors are still at play in most parts of the world and could be worsened by the effects of climate change being experienced.

In as much as intensification of agriculture and biotechnology can offer remedies to food scarcity, the role of wild edible plants cannot be under estimated [14]. They have been reported to make a significant proportion to the global food basket [16]. For instance, in 2014, it was estimated that approximately one billion people globally use wild food plants to supplement their diets [17]. Indeed, in most African communities, the tradition of gathering and consuming wild food plants still persists [9, 15] despite reliance on a few staple crops and huge investments in agriculture [18]. Therefore, it is urgent to recognize the contribution of these plants to food and nutritional security in communities that still use them.

Notwithstanding the contribution of wild edible plants, their diversity and the associated indigenous knowledge (IK) globally have not been documented sufficiently [19]. This situation is worsened by the rampant loss of biological resources [20] and erosion of the associated indigenous knowledge [21, 22]. According to the state of the 2016 world’s plants report, it was estimated that one in every five plants is at risk of extinction globally [23]. In Teso-Karamoja region, it has been reported that 77, 66, and 45% of the natural vegetation cover has been lost in the districts of Katakwi, Kotido, and Kaberamaido districts, respectively [24].

The consumption of wild edible plants in Uganda has been investigated by various scholars in different locales [25–29]. Despite these efforts, it is acknowledged that the diversity of species used is determined by culture and the location [30]. This partly explains why most values of underutilized plants remain undocumented and often not reflected in national and international markets [31, 32]. Consequently, this situation greatly undermines their conservation and sustainable utilization [33]. This ethnobotanical study was warranted due to the information paucity created by cultural and biogeographical diversity, rampant food scarcity [4], and the few botanical surveys in Teso-Karamoja region due to a history of armed conflicts [34]. The study adopted the availability and diversification hypotheses [35], plant use value hypothesis [36], and the versatility hypothesis [37]. These hypotheses helped to generate information pertaining to the diversity of wild edible plant species used, presence or absence of exotics, relative importance of each species and use categories, relative importance of the different lifeforms, seasonal availability, and importance of these plants in the eight forest reserves.

## Materials and methods

### Study area

The study was conducted in eight forest reserves in Teso-Karamoja. They include Onyurut, Bululu Hill, and Ogera Hills (Teso) and Akur, Kano, Mount Napak, Mount Kadam, and Mount Moroto (Moroto). These forest reserves comprise of woodlands, shrublands, and grasslands [24]. They were selected because they are of ecological and biodiversity importance [38]. Secondly, they lacked comprehensive wild edible plant species inventories at the commencement of this study.

Teso sub-region experiences a humid and hot climate with rainfall between 1000 and 1350 mm per annum with the large swamp network playing a moderating role [39]. The sub-region lies at a lower altitude than Karamoja and Sebei sub-regions of Uganda, thereby receiving water discharges which occasionally cause flooding [39]. It is located in the U1 floristic region of the Flora of Tropical East Africa (FTEA) [34]. All the forest reserves herein are predominantly woodlands, bushlands, and grasslands [24].

Karamoja is mainly comprised of semi-arid lands inhabited by pastoralists and agro-pastoralists [3]. It is located in the U3 floristic region of FTEA [34]. The five forest reserves in this sub-region selected for this study are mountainous. All the forest reserves are composed of woodlands, bushlands, and grasslands [24]. However, the South, North, and Eastern escarpment are forested and mountainous areas similar to the well-watered West (Abim district) which has a high vegetation cover comprising of bushlands and woodlands [4]. The lower altitude forests receive variable, unpredictable, and sparse rainfall ranging from 500 to 800 mm per annum while the highlands receive higher amounts. The temperatures are generally high all year round [4].

### Data collection

**Fig. 1:**
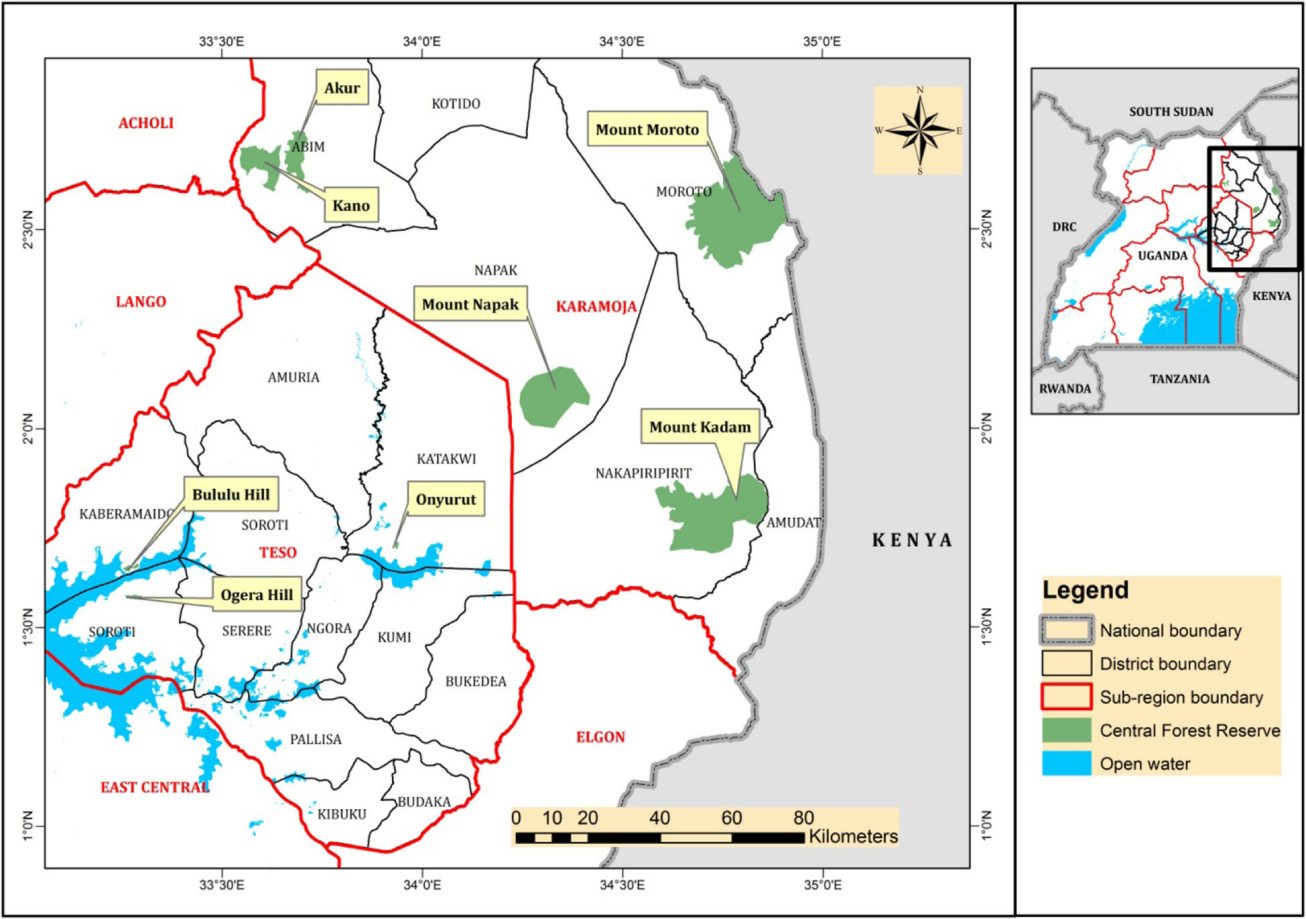
Location of forest reserves in Teso-Karamoja region, Uganda

Data was collected using semi-structured questionnaires administered to 240 respondents living in and around eight forest reserves (Fig. 1) between November 2017 and May 2018. This sample size was determined using Yamane formula for sample size at 95% confidence level [40]. The communities living either in or around each forest reserve were purposely prioritized in the ethnobotanical survey since they have direct interface with the forest reserves. A single village within 1–5 km radius was purposively selected to take part in the survey. The respondents comprised of males and females of different ethnicities, namely Iteso, Kumam, Acholi-Labwor, Tepeth, Bokora, and the Kadam. The first respondent was taken from the first household encountered when approaching a sampling village. Thereafter, systematic simple random sampling was applied until the required sample size was obtained.

The free listing technique [41] was used to capture data on plant identity, the mode of harvesting, consumption mode, and availability patterns. This technique allows the respondents to state the plant name that comes to his/her mind until they are exhausted. In order to corroborate the information gathered in the questionnaires, one focus group discussion with 8–12 respondents was conducted in each forest reserve. These were respondents who stated the highest number of wild edible plant species and were therefore deemed to be more knowledgeable. Field excursions were undertaken with assistance of two male respondents to collect the voucher specimens of the plants enumerated. The voucher specimens were pressed, dried, and identified at the Makerere University Herbarium. The acceptable scientific names were obtained from the catalog of life (http://www.catalogueoflife.org/) while the global conservation status was obtained from the IUCN Red List of Threatened Species (https://www.iucnredlist.org/).

### Data analysis

The data was collated, analyzed using descriptive statistics, and presented using tables and figures. The relative frequency of citation (RFC) was determined for each species as the ratio of respondents who mentioned a particular species to the total number of respondents in the study [42]. In addition, RFC was computed for each use category, lifeform, and season. The RFC values range from 0 to 1 and are a measure of the relative importance. Furthermore, the informants’ consensus factor (FIC) was computed for each used category in order to determine the homogeneity of information given by respondents [43] using a formula FIC = Nur − Nt/(Nur − 1), where Nur is the number of used reports from informants for a particular plant use category and Nt is the number of taxa or species that are used for that plant use category of wild edible plant species.

## Results

### Socio-economic characteristics of respondents

A total of 54% female and 46% male respondents were interviewed. They had varying levels of education whereby 36% had no formal education, 42% primary level, 19% secondary level, and only 3% tertiary level. The respondents comprised of 91% peasant farmers rearing livestock and/or growing crops, 4% petty traders, and 3% fishers while 2% were civil servants.

### Diversity of wild edible plant species

**Fig. 2:**
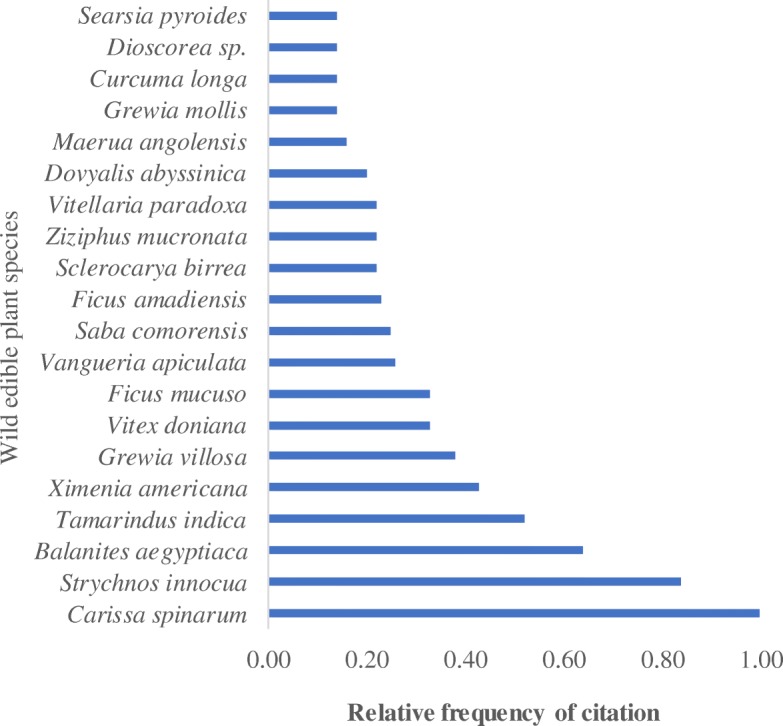
Relative frequency of citation for the top 20 wild edible plant species

**Fig. 3:**
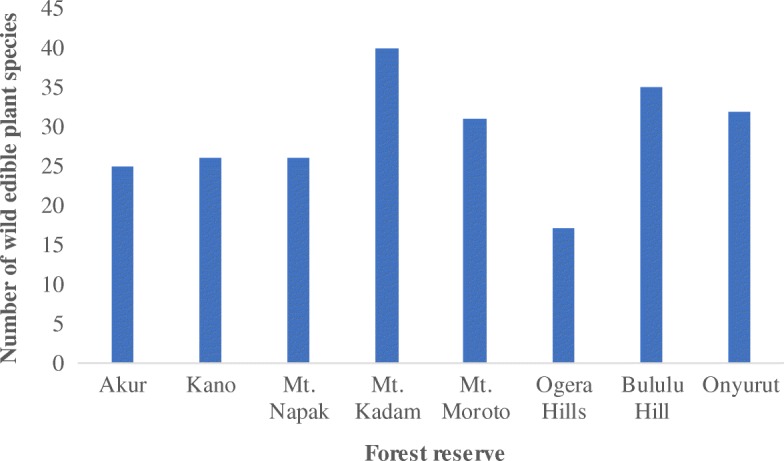
Number of wild edible plant species reported in each forest reserve

This study has established an inventory of 100 wild edible plant species belonging to 47 families in eight forest reserves of Teso-Karamoja region (Table 1). The average number of species mentioned by a respondent was 10 (Table 2). The five plant species with the highest RFC are *Carissa spinarum* L., *Strychnos innocua* Delile, *Balanites aegyptiaca* (L) Delile, *Tamarindus indica* L., and *Ximenia Americana* L. (Fig. 2)*.* The highest number of edible plant species was recorded in Mt. Kadam forest reserve (40) while the lowest was in Ogera hills forest reserve (17) (Fig. 3). There was also a similarity of species across some of the forest reserves with *C. spinarum*, *Vitex madiensis* Oliv., *X. americana*, and *T. indica* occurring in all the reserves. The families Rubiaceae, Fabaceae, Amaranthaceae, Anacardiaceae, and Moraceae had five edible species each.

**Table 1:** Wild edible plant species and their attributes in the eight forest reserves of Teso-Karamoja region, Uganda

Family	Scientific name, authority, and voucher number	RFC	Citation area	Life form	Edible parts	Harvest mode	Cons. state	Season	IUCN GCS
Acanthaceae	*Justicia flava* (Forssk.) Vahl (OJ 185)	0.03	8	Forb	Fr	Plu	Ra	Rainy	NE
	*Asystasia mysorensis* (Roth) Anders.(OJ 26)	0.03	1	Forb	Ve	Plu	Co	Rainy	NE
Aloaceae	*Aloe* sp. (OJ 106)	0.01	5	Forb	Ve	Plu	Ra	Rainy	–
Amaranthaceae	*Psilotrichum axilliflorum* Suess.(OJ 10)	0.13	1	Shrub	Fr	Plu	Ra	Rainy	EN
	*Amaranthus spinosa* L.(OJ 37)	0.01	2	Forb	Ve	Plu	Co	Rainy	NE
	*Amaranthis hybridus* L. subsp. *cruentus* (L) Thell (OJ 125)	0.01	6	Forb	Ve	Plu	Co	Rainy	NE
	*Amaranthus graecizans* L.(OJ 126)	0.01	6	Forb	Ve	Plu	Co	Rainy	NE
	*Amaranthus spinosus* L. (OJ 127)	0.01	6	Forb	Ve	Plu	Co	Rainy	NE
	*Cloeme* sp. (OJ 24)	0.03	1	Forb	Ve	Plu	Co	Dry	NE
Anacardiaceae	*Mangifera foetida* Lour. (OJ 18)	0.16	1,2,3	Tree	Fr	Plu/Col	Ra	Rainy	DD
	*Sclerocarya birrea* (A. Rich) Hochst. (OJ 58)	0.23	2,3,4,5,7	Tree	Fr	Col/Plu	Ra	Rainy	NE
	*Searsia pyroides* (Burch.) Moffett (OJ 38)	0.17	2,3	Shrub	Fr	Plu	Ra	Rainy	NE
	*Searsia ruspolii* (Engl.) Moffett (OJ 81)	0.17	4,5,6,7,8	Shrub	Fr	Plu	Ra	Dry	NE
	*Toxicodendron rydbergii* (Small ex Rydb.) Greene (OJ 39)	0.06	2	Shrub	Fr	Plu	Ra	Rainy	NE
Annonaceae	*Monanthotaxis buchananii* (Engl.) Verdc (OJ 36)	0.12	2	Shrub	Fr	Plu	Ra	Dry	NE
	*Annona senegalensis* Pers.(OJ 53)	0.09	2,3,4,5,6,7,8	Shrub	Fr	Plu	Ra	Rainy	NE
Apocynaceae	*Leptadenia hastata* (Schumach. & Thonn.) Decne (OJ 3)	0.06	1	Climber	Ve	Plu	Co	Dry	NE
	*Carissa spinarum* L.(OJ 4)	1.00	1,2,3,4,5,6,7,8	Shrub	Fr	Plu	Ra	Rainy	NE
	*Ceropegia johnstonii* (N.E.Br.) Bruyns (OJ 78)	0.10	4,5,6	Forb	Tu	Dig	Co/Ra	Dry	NE
	*Saba comorensis* (Bojer) Pichon (OJ 139)	0.25	4,6,7,8	Tree	Fr	Plu	Ra	Rainy	NE
	Scientific name and voucher number								
Araceae	*Colocasia esculenta* (L.) Schott (OJ 57)	0.01	2	Forb	Tu	Dig	Co	All	LC
Arecaceae	*Borassus aethiopum* Mart. (OJ 63)	0.04	2,7,8	Tree	Fr	Col/Plu	Ra	Dry	LC
Asparagaceae	*Asparagus flagellaris* (Kunth) Baker (OJ 52)	0.13	2	Forb	Ve	Plu	Co	All	NE
Asteraceae	*Lactuca inermis* Forsk. (OJ 204)	0.04	1	Forb	Ve	Plu	Co	Rainy	NE
Basellaceae	*Basella alba* L.(OJ 129)	0.10	4	Cimber	Ve	Plu	Co	Rainy	NE
Brassicaceae	*Arabis alpina* L. (OJ 27)	0.01	1	Forb	Ve	Plu	Co	Rainy	NE
Cactaceae	*Opuntia monacantha* (Willd.) Haw.(OJ 111)	0.01	5	Tree	Fr	Plu	Ra	Rainy	NE
Capparaceae	*Maerua angolensis* DC.(OJ 2)	0.20	1,3	Shrub	Ve	Plu	Co	Dry	NE
	*Capparis fascicularis* DC. (OJ 74)	0.09	5,6	Shrub	Ve	Plu	Co	Dry	NE
Caricaceae	*Carica papaya* L.(OJ 42)	0.10	2	Tree	Fr	Plu	Ra	All	DD
Celastraceae	*Catha edulis* Forsk.(OJ 123)	0.06	6	Shrub	Ve	Plu	Ra	Rainy	LC
Cucurbitaceae	*Cucurbita* sp. (OJ 51)	0.06	2	Climber	Fr/Ve	Plu	Co	All	–
	*Momordica foetida* Schum.(OJ 82)	0.01	1,2,3	Forb	Ve	Plu	Co	Rainy	NE
	*Cucumis ficifolius* A. Rich.(OJ 44)	0.06	5,7	Climber	Fr	Plu	Co	Dry	NE
Dioscoreaceae	*Dioscorea* sp.(OJ 176)	0.19	7,8	Climber	Tu	Dig	Ra	Rainy	–
	*Dioscorea sagittifolia* var. *lecardii* (De Wild.) Nkounkou (OJ 72)	0.10	4,5,6	Climber	Tu	Dig	Co	Dry	NE
	*Dioscorea bulbifera* L.(OJ 41)	0.04	2	Climber	Tu	Dig	Co	All	NE
Ebenaceae	*Diospyros mesipiliformis* A.DC.(OJ 6)	0.07	1,2,5,6,7,8	Tree	Fr	Plu	Ra	Dry	NE
	*Diospyros* sp. (OJ 21)	0.13	1	Tree	Fr	Plu	Ra	Rainy	–
	*Euclea divinorum* Hiern (OJ 113)	0.03	5,6	Tree	Fr	Plu	Ra	Rainy	NE
	*Diospyros abyssinica* (Hiern) F.White (OJ 94)	0.06	5,6	Tree	Se	Col	Ra	dry	NE
Ehretiaceae	*Cordia monoica* Roxb.(OJ 174)	0.01	4	Tree	Fr	Col/Plu	Ra	Rainy	NE
Euphorbiaceae	*Bridelia scleroneura* Müll. Arg. (OJ 62)	0.13	1,2,3,4,5,6,7,8	Tree	Fr	Plu	Ra	Rainy	NE
Fabaceae	*Tamarindus indica* L. (OJ 8)	0.52	1,2,3,4,5,6,7,8	Tree	Fr	Plu	Co/Ra	Dry	LC
	*Vigna kirkii* (Baker) J.B.Gillett (OJ 25)	0.03	1,4,5,6	Climber	Ve	Plu	Co	Dry	NE
	*Rhynchosia goetzei* Harms (OJ 114)	0.01	5	Shrub	Se	Col	Co	Rainy	NE
	*Senegalia senegal* (L.) Britton (OJ 68)	0.01	5	Tree	Gu	Plu	Ra	Dry	NE
	*Senna obtusifolia* (L.) H.S.Irwin & Barneby (OJ 20)	0.03	1,2,7,8	Forb	Ve	Plu	Co	All	NE
Hydnoraceae	*Hydnora abyssinica* A.Br.(OJ 80)	0.14	4,5,6	Shrub	Ve	Plu	Co	Rainy	NE
Lamiaceae	*Hoslundia opposita* Vahl (OJ 30)	0.03	2,6,7,8	Forb	Fr	Plu	Ra	Dry	NE
	*Vitex madiensis* Oliv. (OJ 12)	0.14	1,2,3,4,5,6,7,8	Tree	Fr	Plu	Ra	Rainy	NE
	*Ocimum gratissimum* L. (OJ 197)	0.01	7	Forb	Fr	Plu	Ra	Rainy	NE
	*Vitex doniana* Sweet (OJ 39)	0.38	2,7,8	Shrub	Fr	Plu	Ra	Rainy	LC
Loganiaceae	*Strychnos spinosa* Lam. (OJ 34)	0.06	4,5,6	Shrub	Fr	Col/Plu	Ra	Dry	NE
	*Strychnos innocua* Delile (OJ 33)	0.84	1,2,3,7,8	Shrub	Fr	Col/Plu	Ra	Dry	NE
	*Strychnos* sp.(OJ 150)	0.01	6	Tree	Fr/Se	Plu/Col	Ra	Dry	–
Malvaceae	*Hibiscus cananabinus* L.(OJ 28)	0.03	1,6	Forb	Ve	Plu	Co	Rainy	NE
	*Hibiscus acetosella* Welw. ex Fic. (OJ 166)	0.01	4,5,6	Shrub	Fr	Plu	Ra	Rainy	NE
	*Sterculia setigera* Del. (OJ 198)	0.04	7,8	Tree	Se	Col	Ra	Rainy	NE
	*Grewia trichocarpa* Hochst. ex A. Rich.(OJ 192)	0.03	7,8	Tree	Fr	Plu	Ra	Rainy	NE
Moraceae	*Ficus mucuso* Welw. ex Ficalho (OJ 73)	0.33	4,5,6,7,8	Tree	Fr	Plu	Ra	Rainy	NE
	*Ficus natalensis* Hochst. (OJ 65)	0.01	4,5,6,7,8	Tree	Fr	Plu	Ra	Rainy	NE
	*Ficus platyphylla* Del.(OJ 86)	0.03	4,5,6,7,8	Tree	Fr	Plu	Ra	Rainy	NE
	*Ficus ovata* Vahl (OJ 96)	0.01	4,5,6,7,8	Tree	Se	Col	Ra	Rainy	NE
	*Ficus thonningii* Bl.(OJ 185)	0.06	7,8	Tree	Fr	Plu	Ra	Rainy	NE
	*Ficus amadiensis* De Wild.(OJ 105)	0.23	4,5,6	Tree	Fr	Plu	Ra	Rainy	NE
	*Ficus ingens* (Miq.) Miq.(OJ 189)	0.10	7,8	Tree	Ve	Plu	Co	Rainy	NE
Musaceae	*Musa paradisiaca* L. (OJ 187)	0.01	7,8	Forb	Fr	Plu	Ra	Rainy	NE
Myrtaceae	*Psidium guajava* L. (OJ 64)	0.01	2	Shrub	Fr	Plu	Ra	Rainy	NE
Papilionaceae	*Crotalaria* sp. (OJ 199)	0.03	1	Forb	Ve	Plu	Co	Rainy	NE
Passifloraceae	*Passiflora edulis* Sims (OJ 32)	0.10	2	Climber	Fr	Col/Plu	Ra	Rainy	NE
Pedaliaceae	*Sesamum angustifolium* (Oliv.) Engl (OJ 29)	0.03	1,2	Forb	Ve	Plu	Co	Rainy	NE
Poaceae	*Oxytenanthera abyssinica* (A.Rich.) Munro (OJ 180)	0.01	7,8	Grass	Ve	Plu	Co	Rainy	NE
Polygonaceae	*Oxygonum sinuatum* (Hochst. & Steud. ex Meisn.) Damm. (OJ 200)	0.03	1	Forb	Ve	Plu	Co	Rainy	NE
Rhamnaceae	*Ziziphus mucronata* Willd. (OJ 179)	0.22	1,4,5,6,7,8	Shrub	Fr	Plu	Ra	Dry	NE
Rubiaceae	*Catunaregam nilotica* (Stapf) Tirveng. (OJ 9)	0.16	1,2,3	Shrub	Fr	Plu	Ra	Rainy	NE
	*Mitragyna stipulosa* (DC.) Kuntze (OJ 184)	0.01	7,8	Tree	Fr	Col	Ra	Rainy	VU
	*Canthium lactescens* Hierm (OJ 11)	0.10	1,6	Shrub	Fr	Plu	Ra	Rainy	NE
	*Gardenia ternifolia* Schumach. & Thonn.(OJ 146)	0.03	6	Tree	Fr	Plu	Ra	Dry	NE
	*Vangueria apiculata* K.Schum. (OJ 104)	0.26	1,4,5,6,7,8	Tree	Fr	Plu	Ra	Rainy	NE
	*Rytigynia neglecta* (Hiern) Robyns (OJ 107)	0.01	5	Tree	Fr	Plu	Ra	Rainy	NE
Rutaceae	*Zanthoxylum leprieurii* Guill. & Perr.(OJ 23)	0.04	1,5	Tree	Fr	Plu	Co	Dry	NE
Salicaceae	*Dovyalis abyssinica* (Rich.) Warb. (OJ 67)	0.20	4,5,6	Shrub	Fr	Plu	Ra	Rainy	NE
	*Oncoba spinosa* Forssk (OJ 201)	0.06	4,5,6	Shrub	Fr	Pl	Ra	Rainy	NE
Sapindaceae	*Allophylus rubifolius* (Hochst. ex A. Rich.) Engl.(OJ 15)	0.07	1	Shrub	Fr	Plu	Ra	Rainy	NE
Sapotaceae	*Vitellaria paradoxa* C.F.Gaertn. (OJ 7)	0.22	1,3,4,5,6,7,8	Tree	Fr/Se	Col	Co/Ra	Rainy	VU
Solanaceae	*Physalis lagascae* Roem. & Schult. (OJ 19)	0.01	1	Forb	Fr	Plu	Ra	Dry	LC
	*Physalis peruviana* L.(OJ 59)	0.06	2	Forb	Fr	Plu	Ra	Rainy	NE
	*Solanum lycopersicum* L. (OJ 31)	0.09	2	Forb	Fr	Plu	Co	Rainy	NE
	*Capsicum frutescens* L.(OJ 35)	0.07	2	Forb	Fr	Plu	Co/Ra	All	LC
Tiliaceae	*Grewia villosa* Willd (OJ 17)	0.38	1,4,5,7,8	Shrub	Fr	Plu	Ra	Dry	NE
	*Grewia mollis* Juss. (OJ 22)	0.19	1,2,3,4,5,6,7,8	Shrub	Fr	Plu	Ra	Rainy	NE
Verbanaceae	*Lantana camara* L.(OJ 47)	0.06	2	Shrub	Fr	Plu	Ra	Rainy	NE
	*Lippia abyssinica* (Otto & A.Dietr.) Cufod.(OJ 182)	0.03	8	Forb	Se	Plu	Ra	Rainy	NE
Vitaceae	*Cyphostemma cyphopetalum* (Fresen.) Desc.(OJ 202)	0.07	1,2,4,5,6,7,8	Climber	Ve	Plu	Co	All	NE
Ximeniaceae	*Ximenia americana* L. (OJ 5)	0.43	1,2,3,4,5,6,7,8	Tree	Fr	Plu	Ra	Dry	NE
Zingiberaceae	*Curcuma longa* L. (OJ 55)	0.19	2,3,7,8	Forb	Fr	Plu	Ra	Dry	NE
Zygophyllaceae	*Balanites aegyptiaca* (L.) Delile (OJ 1)	0.64	1,2,3,4,5,6,7,8	Tree	Fr/Ve	Plu/Col	Co/Ra	Dry	NE
	*Tribulus terrestris* L. (OJ 203)	0.01	6	Forb	Ve	Plu	Co	Rainy	NE
	*Balanites rotundifolia* (van Tiegh.) Blatter (OJ 164)	0.03	4,5	Tree	Fr	Plu	Ra	Dry	NE

Key:

1. RFC-relative frequency of citation

2. Citation area-central forest reserve where the wild edible plant was collected:1-Onyurut, 2-Bululu Hill, 3-Ogera Hill, 4-Mount Napak, 5-Mount Moroto, 6-Mount Kadam, 7-Akur, and 8-Kano

3. Edible parts: Fr-fruits, Ve-vegetables, Se-seeds, Gu-gum, and Tu-tuber

4. Harvest mode-harvesting mode: Plu-plucking from mother plant, Col-collecting from the ground, Dig-digging

5. Cons. state-consumption state: co-requires cooking, Ra-eaten raw

6. IUCN GCS-IUCN Global Conservation Status: EN-endangered, VU-vulnerable, LC-least concern, DD-data-deficient and NE-not evaluated

**Table 2:** Average wild edible species by respondents and use category

Forest reserve name	Species per respondent	Species per use category				
		Fruits	Vegetables	Tubers	Seeds	Gum
Akur	12	8.89	1.22	0.67	0.33	0.00
Kano	8.4	7.18	0.18	1.45	0.36	0.00
Ogera Hills	5.3	5.00	0.27	0.00	0.13	0.00
Mount Kadam	19.2	14.50	2.67	0.67	0.67	0.17
Mount Napak	9.3	8.00	0.50	2.80	0.20	0.10
Mount Moroto	9.7	8.40	0.73	0.50	0.00	0.00
Bululu Hills	9.6	8.07	0.87	0.29	0.29	0.00
Onyurut	7.2	4.20	2.64	0.07	0.29	0.00
Overall average	10.1	8.03	1.14	0.81	0.29	0.03

### Lifeforms of wild edible plant species

**Fig. 4:**
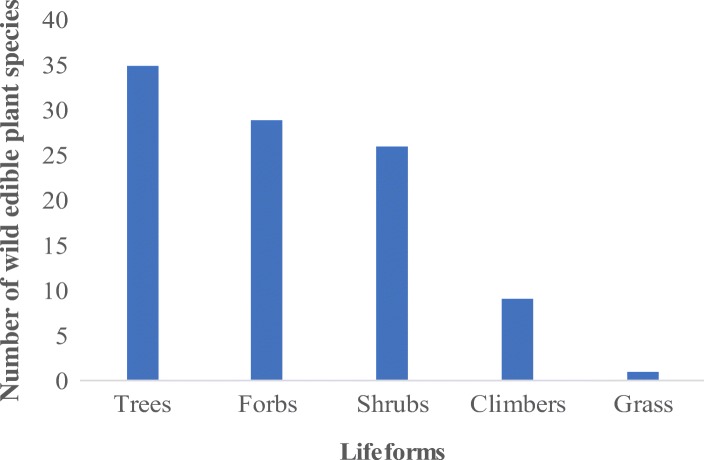
Lifeforms of wild edible plant species in the forest reserves of Teso-Karamoja

**Fig. 5:**
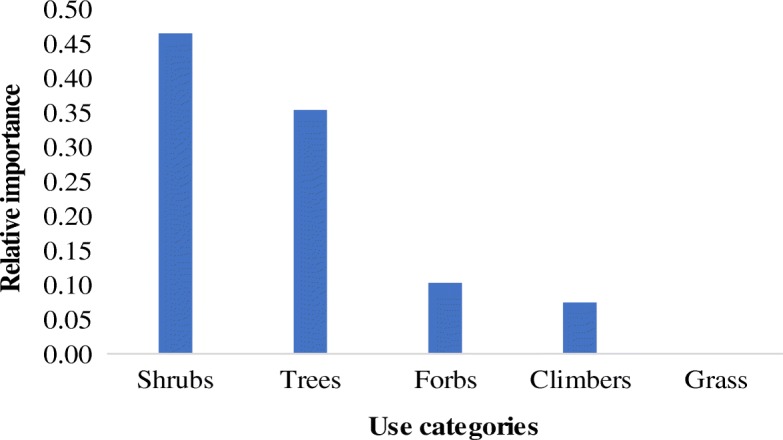
Relative importance of the lifeforms of wild edible plant species in forest reserves of Teso-Karamoja

The wild edible plant species belong to five lifeforms namely grass, forbs, shrubs, trees, and climbers. Trees contributed 35% while the grasses contributed only 1.0% of all the identified species (Fig. 4). It is further noted that the shrubs are the most important source of wild food (RFC = 0.47) while grass are the least (RFC = 0.00) (Fig. 5).

### Use categories of wild edible plant species

**Fig. 6:**
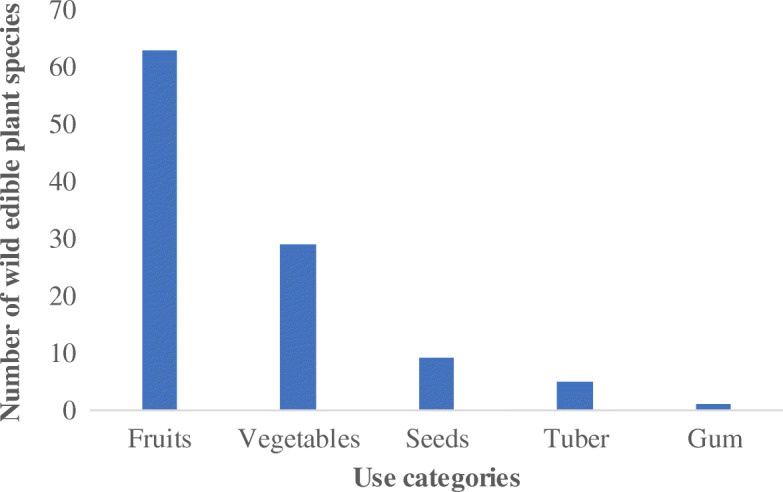
Use categories of wild plant species in Teso-Karamoja, Uganda

**Fig. 7:**
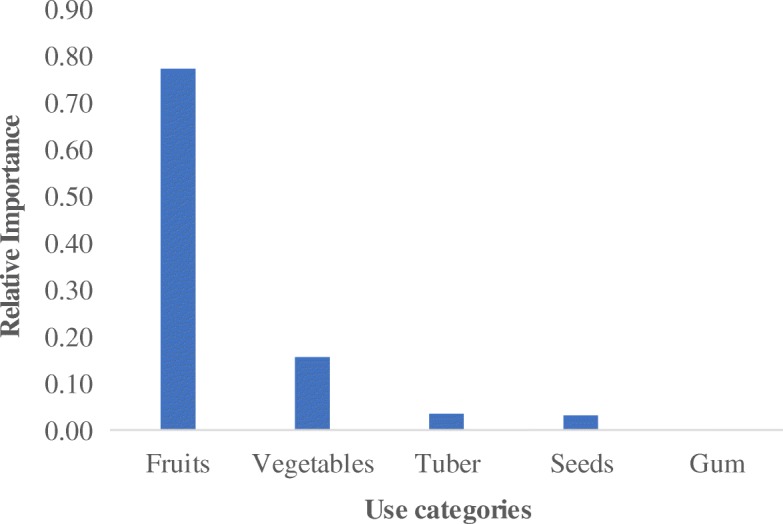
Relative importance of the use categories of wild edible plant species

Five use categories of wild edible plants have been documented in Teso-Karamoja region. These are fruits, vegetables, seeds, tubers, and gum (Fig. 6). Fruits were provided by 63% of the species while those providing gum comprised 1%. Most of species provide food that is eaten without cooking (64%) while the reminder (34%) requires cooking. Some of the species eaten without cooking are fruits from *C. spinarum*, *Psilotrichum axilliflorum* Suess, and *V. doniana.* Respondents agreed that fruits were the most important use value of wild edible plants (RFC = 0.78) (Fig. 7). The relative importance of fruits, vegetables, and gum concurs with the prominence of species consumed in each category except for tubers which are relatively more important than seeds. There was no respondent homogeneity in the use of gum for food whilst it was almost homogenous for seeds (RFC = 0.93) (Table 3).

**Table 3:** Factor of informants’ consensus (FIC) for each use category

Categories	Number of taxa (Nt)	Number of use report (Nur)	Informant’s consensus factor (FIC)
Fruits	61	595	0.90
Vegetables	27	124	0.79
Tubers	05	19	0.80
Seeds	06	26	0.93
Gum	01	01	0.0

### Seasonality of wild edible plant species

The seasons in Teso-Karamoja region are broadly divided into dry (November to March) and rainy (April to October). The species used throughout the year comprise 9%, rainy season 64%, and dry season 27% (Table 1). The main plants in the dry season include *T. indica* and *B. aegyptiaca* while the rainy season species includes *C. spinarum*, *V. paradoxa*, and *V. madiensis.* It was observed that members of the community preserved and stored some of the plants in order to guarantee supply during the off-peak seasons. The species mentioned include *T. indica*, *V. paradoxa*, and *Dioscorea* sp. The relative seasonal importance of the wild edible plant species shows that they are more important in the rainy season (RFC = 0.55) than in the dry season (RFC = 0.40) and throughout the year (RFC = 0.05).

### Preference of wild edible plant species

The consumption of wild edible plant species was premised on five reasons, namely (i) hunger due to food scarcity, (ii) spicing staple food, (iii) preservation of cultural practice, (iv) nutri-medicinal value, and (v) their delicacy. *Zanthoxylum leprieurii* Guill. & Perr was commonly mentioned nutri-medicinal plant for flavoring tea but also used in treating various ailments.

### Harvesting techniques

Wild edible plants were mainly harvested using three rudimentary methods, namely digging (tubers and roots), plucking from plants (fruits, seeds, and gum), and ground collection of fallen seeds and fruits. The prominence of these techniques was in the order of plucking from mother plants (82%), collecting from the ground (13%), and digging (5%).

### Global conservation status of wild edible plant species

**Fig. 8:**
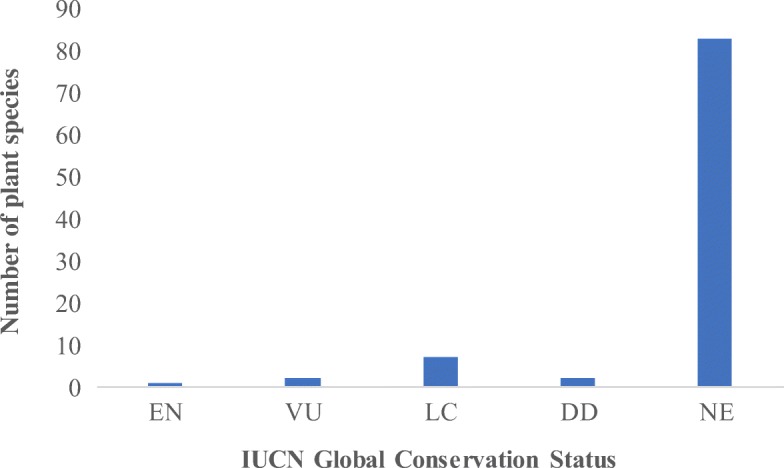
IUCN Global Conservation Status of wild edible plant species

The only globally threatened plant species recorded in this study are *P. axilliflorum* (endangered), *Mitragyna stipulosa* (DC.) Kuntze (Vulnerable), and *Vitellaria paradoxa* C.F.Gaertn (vulnerable). The rest are either least concern (LC), data deficient (DD), or not evaluated (NE) (Fig. 8). The voucher specimens of five plants could not be identified up to the species level and were ultimately not assigned to any of the IUCN categories.

## Discussion

The high diversity of wild edible plant species (Table 1) in Teso-Karamoja region demonstrates that people in and around forest reserves possess information about local vegetation that provides food. This is in tandem with the availability hypothesis [35] which asserts that people use plants that are more accessible or locally abundant. It ought to be noted that availability is often conceptualized as a physical distance from a home or community to the location where the plant grows in the wild but can also be considered in terms of seasonality, abundance, and price as well as access to markets, gardens, or natural areas where the plants are found [35]. The number of species recorded in this study is comparable to the 114 edible plant species (57 families) reported in the Eastern Arc Mountains of Tanzania [6]. However, it is also higher than the 77 species reported in Ethiopia [44], 72 species in Kenya [45], and 52 species reported in the Middle Agri Valley of Italy [46]. This variance in the diversity of edible wild plant species has been attributed to disparities in culture and location [30]. The Rubiaceae, Fabaceae, Anacardiaceae, Amaranthaceae, and Moraceae families are widely known to be among the largest and economically important sources of food and are widespread in the tropics [9].

The presence of exotic species namely *Passiflora edulis* Sims., *Carica papaya* L., *Solanum lycopersicum* L., *Lantana camara* L., *Musa paradisiaca* L., *Psidium guajava* L., and *Amaranthis hybridus* L. subsp. *cruentus* (L) Thell among the wild edible plants in this region is evidence for the diversification hypothesis [35]. The incorporation of exotic plants in traditional diets enriches culture as opposed to being a sign of cultural erosion or environmental degradation. These exotics exist as weedy escapees, naturalized, or invasive species while some of them such as *P. edulis*, *P. guajava*, *C. papaya*, *A. hybridis*, *M. paradisiaca*, *and S. lycopersicum* can be purposively cultivated.

All the forest reserves investigated are woodlands, and this partly explains why the trees represent the majority of edible plant species (Fig. 4), they are not the most important sources of wild food (Fig. 5). This information is very useful in prioritizing wild plant species for domestication initiatives or in situ conservation and further investigation of their nutritional and mineral quality. Again, the number of species in each lifeform is dynamic and varies from one ecological niche to another. While herbs were the majority amongst the subsistence farming communities of Amuria district, Uganda [47], in Nhema Communal Area, Midlands Province, Zimbabwe, trees comprised the majority of edible species [9]. These results conform to the plant use value hypothesis proposition that the usefulness of a plant for food, medicine, construction, technology, or trade in any community is directly related to its botanical family, lifeform, local abundance, and/or maximum size [36].

The fruits were considered the most important use category (Fig. 7) because they require no sophisticated means of preparation. The average number of wild edible plant species in Table 2 affirms the relative importance of the five use categories in Fig. 7. They are often eaten opportunistically as one is undertaking other core activities such as gardening, grazing, collecting firewood, fetching water, or hunting. Whereas opportunistic collection of wild edible plants was attributed to only women and children during firewood or water fetching [48], our findings reveal that even adult males engaged in hunting, livestock grazing, gardening, and collecting construction materials opportunistically forage wild edible plants. Generally, the fondness of an edible part is attributable to the ease of processing, nutritional value, and the taste [18].

**Plate 1:**
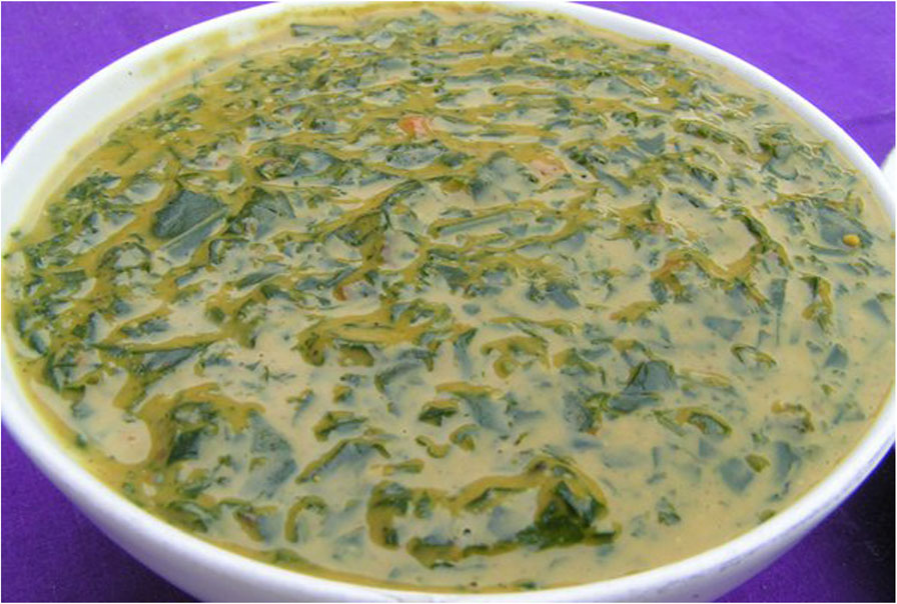
Sour milk, groundnut, or simsim paste are added as spice

The procedure for preparing *V. kirkii* (Baker) J.B.Gillett and *S. obtusifolia* (L.) H.S.Irwin & Barneby vegetable sauce is similar, namely plucking fresh leaves, brief wilting under direct sunlight for approximately 30 min, washing, and then boiling. In order to ensure proper cooking, local salt called “*Abalang*” (filtrate from ash of selected plants) is added, and then, sodium chloride is added to give a good taste. This can be eaten at this stage, or sour milk, groundnut, or simsim paste are added to spice it (Plate 1). The peculiarity in *Maerua angolensis* DC, and *L. hastata*, is pounding (using a mortar and pestle) after boiling and no addition of the local salt. In the case of *Dioscera* sp. tubers, they are washed (occasionally peeled), boiled, and salted (sodium chloride), and it is ready to be eaten.

According to the optimal foraging theory [10], the results obtained in this study imply that the most economically advantageous foraging pattern is eating fruits. This theory asserts that although obtaining food provides an individual with energy, searching and capturing it also requires energy and time. This underpinning ably explains why adults interviewed abandoned the consumption of certain plants such as *S. innocua* and *S. birrea* to children although they are familiar with them but the energy and time invested in obtaining them is not commensurate with the resultant value. This tendency has also been reported in Zimbabwe [9, 48]. Generally, humans contemplate the choice of their food by considering its nutritional benefit together with the cost of searching, handling, and collecting it [49].

The lack of respondent agreement on the use of gum as food (Table 3) is largely because it is only chewed without swallowing in worse-case scenarios of hunger. It is also probable that this observation is an outlier. However, the loss of indigenous knowledge about edible wild plants [50] change in the livelihood patterns that limit the amount of time spent foraging in the wild [51] and a general shift from gathered to purchased foods as rural communities join the market economy [52] could be responsible for this scenario. Whereas the gatherer to purchaser scenario exists in Teso-Karamoja, the most applicable scenario could be described as gatherer to purchased, cultivated, and sometimes donated (relief) food.

The relatively high importance of wild edible plants in the rainy season coincides with the time when most species are re-sprouting, flowering, and fruiting, thereby increasing their availability. It ought to be stressed that during the rainy season, most households are able to produce food from a range of crops and therefore have a wide range of choice, but still wild edible plants are important. In the dry season, communities are solely dependent on stored food and the wild edible plants (especially vegetables) help to diversify the food intake. This seasonal relative importance greatly impacts households’ food and nutritional insecurity copying ability. Earlier scholars have asserted that in times of food scarcity, wild edible plants make human diets more diverse and add flavor, vitamins, and minerals [53]. Globally, wild edible plants have been recognized as a key component in ecosystem-based adaptation [5] and food scarcity copying strategy [2].

The factors put forward for using wild edible plants in Teso-Karamoja region exemplify the vital role they play in the community. Therefore, even if every rural household had enough food in this region, the use of wild edible plants would still exist. There is a need to ensure that the biological resources and the associated indigenous knowledge are safeguarded for posterity, where both the biological resources and the cultural heritage of these communities shall be preserved. It has also been established that wild edible plants harbor great cultural significance to rural populations in developing countries [6] and their attachment to culture partly explains why the ancient hunter-gatherer tradition still persists in some African communities [9, 15]*.* This conforms to the proposition of the versatility hypothesis that people are more likely to retain their knowledge and the use and access to a plant that has a greater value for humans [37].

All the methods used to harvest wild edible plants in this region can be termed as rudimentary and therefore pose less deleterious effects to the plant species. However, where they involve cutting of branches in order to pluck off edible vegetables such as in *B. aegyptiaca* and *M. angolensis*, the growth and survival of the plant is greatly hampered. Another deleterious example is the collection of the tubers by digging. These cases imply that forest reserve management plans ought to consider regulated collection of such peculiar plants in order to minimize any detrimental effects. Awareness that most rural communities in Teso-Karamoja region (Uganda generally) feel aggrieved by most forest conservation measures, streamlining the collection of wild edible plants as a trade-off could be a step towards garnering the support of these communities.

The most threatened plant species encountered in this study is *P. axilliflorum.* This is an endangered species which is endemic to the Democratic Republic and Congo (DRC) and Uganda [54]. In Uganda, it was only recorded in Budongo Central Forest Reserve and Lake Mburo National Park [54]. The current finding is therefore an important step in improving the understanding of the species’ biogeography. Almost all the wild edible plant species in this area have uncertain conservation status (not evaluated) (Fig. 8), yet it is generally accepted that lack of suitable data for prioritizing conservation action greatly hampers plant conservation efforts [55]. However, it should be noted that some of the taxa in the not evaluated category are cosmopolitan species. Some of them are in the family Amaranthaceae which constitute most weeds and secondary crops occurring abundantly in different ecological areas. *T. indica* has been assessed as vulnerable by the National Red List of Uganda [56] but has not been assessed by the IUCN.

The wild edible plants in Teso-Karamoja region ultimately offer various benefits and opportunities to the dependent communities. These plants present a cheap means of enriching the diets, creating employment, and diversifying the livelihoods of rural communities in the rural communities of Teso-Karamoja region. There are already some isolated individuals and trade companies exploiting the collection of gum Arabic from *Senegalia senegal* (L.) Britton, shea nuts from *V. paradoaxa*, tamarinds from *T. indica*, and desert date from *B. aegyptiaca*. Other initiatives include making of wine from *S. innocua*, *B. schleronuera*, *V. madiensis*, and *C. spinarum.* However, the challenge is that these initiatives are highly fragmented, uncoordinated, and poorly documented, thereby preventing the realization of their full potential.

## Conclusion

A high diversity of wild edible plant species in the forest reserves of Teso-Karamoja region has been established. The majority of species are natives with a limited range of exotics. Fruit consumption is the most important use category of wild edible plants in this area. All the species enumerated are considered important throughout the year because they serve different purposes. Therefore, this study has certainly made a significant contribution to the preservation of the cultural heritage in a form of wild edible plant indigenous knowledge in this region.

## Recommendations

There is a need to store the documented information in user-friendly formats for access by the local communities. The development of propagation protocols for highly rated species will go along in promoting a deliberate domestication drive, thereby reducing the dependence on the wild population. It is also pertinent to elucidate the nutritional contribution of these plants to human diets and explore their role as trade-offs in forest reserve management.
